# Gut microorganisms of *Locusta migratoria* in various life stages and its possible influence on cellulose digestibility

**DOI:** 10.1128/msystems.00600-24

**Published:** 2024-06-18

**Authors:** Kai Li, Wen-Jing Li, Ke Liang, Fei-Fei Li, Guo-Qing Qin, Jia-Hao Liu, Yu-Long Zhang, Xin-Jiang Li

**Affiliations:** 1 The Key Laboratory of Zoological Systematics and Application, School of Life Sciences, Institute of Life Sciences and Green Development, Hebei University, Baoding, China; University of California San Diego, La Jolla, California, USA

**Keywords:** *Locusta migratoria*, gut microbial diversity, life stages, cellulose digestibility, cellulolytic bacteria

## Abstract

*Locusta migratoria* is an important phytophagous pest, and its gut microbial communities play an important role in cellulose degradation. In this study, the gut microbial and cellulose digestibility dynamics of *Locusta migratoria* were jointly analyzed using high-throughput sequencing and anthrone colorimetry. The results showed that the gut microbial diversity and cellulose digestibility across life stages were dynamically changing. The species richness of gut bacteria was significantly higher in eggs than in larvae and imago, the species richness and cellulose digestibility of gut bacteria were significantly higher in early larvae (first and second instars) than in late larvae (third to fifth instars), and the diversity of gut bacteria and cellulose digestibility were significantly higher in imago than in late larvae. There is a correlation between the dynamics of gut bacterial communities and cellulose digestibility. *Enterobacter*, *Lactococcus,* and *Pseudomonas* are the most abundant genera throughout all life stages. Six strains of highly efficient cellulolytic bacteria were screened, which were dominant gut bacteria. Carboxymethyl cellulase activity (CMCA) and filter paper activity (FPA) experiments revealed that *Pseudomonas* had the highest cellulase enzyme activity. This study provides a new way for the screening of cellulolytic bacteria and lays the foundation for developing insects with significant biomass into cellulose-degrading bioreactors.

**IMPORTANCE:**

Cellulose is the most abundant and cheapest renewable resource in nature, but its degradation is difficult, so finding efficient cellulose degradation methods is an urgent challenge. *Locusta migratoria* is a large group of agricultural pests, and the large number of microorganisms that inhabit their intestinal tracts play an important role in cellulose degradation. We analyzed the dynamics of *Locusta migratoria* gut microbial communities and cellulose digestibility using a combination of high-throughput sequencing technology and anthrone colorimetry. The results revealed that the gut microbial diversity and cellulose digestibility were dynamically changed at different life stages. In addition, we explored the intestinal bacterial community of *Locusta migratoria* across life stages and its correlation with cellulose digestibility. The dominant bacterial genera at different life stages of *Locusta migratoria* were uncovered and their carboxymethyl cellulase activity (CMCA) and filter paper activity (FPA) were determined. This study provides a new avenue for screening cellulolytic bacteria and lays the foundation for developing insects with significant biomass into cellulose-degrading bioreactors.

## INTRODUCTION

Grasshoppers (Orthoptera: Acridoidea) are migratory pests that damage grain crops such as wheat and maize, as well as cash crops such as cotton. Their excellent reproductive ability, rapid migration, and dispersal make them a major global economic threat. *Locusta migratoria* and *Schistocerca gregaria* are not only highly dangerous but also valuable model research insects for studying various aspects of insect life, including morphology, physiology, ecology, behavior, and neurology (1). *L. migratoria* primarily feeds on graminaceous plants. Within their digestive system, gut microorganisms degrade and ferment cellulose and hemicellulose, which are the main components of plant cell walls, resulting in the production of volatile fatty acids, serving as the primary source of nutrients for locusts (2). Cellulose and hemicellulose are major components of plant biomass and play a crucial role in driving microbial and animal heterotrophy. The significant feeding capacity and robust digestive ability of *L. migratoria* provide valuable insights for enhancing cellulose and hemicellulose degradation in engineered biomass conversion sites, such as waste treatment plants and biorefineries.

Insect gut microbial communities co-evolve with their hosts, enabling insects to adapt to different diets and overcome the limitations of obtaining nutrients from food and countering defensive chemicals (3). Insect guts commonly harbor bacterial families such as Enterobacteriaceae, Pseudomonadaceae, and Lactobacillaceae, which play vital roles in digestion, immunity, detoxification, and reproduction processes (4–6). For instance, in *Drosophila*, Enterobacteriaceae aids in digestion and absorption by degrading carbohydrates in the intestinal tract (7). Gut bacteria in herbivorous turtle ants recycle urea and uric acid to provide essential amino acids to the host (8). In black soldier flies, *Bacillus* contributes to protein accumulation through high levels of amino acid metabolism (9). *Pantoea* helps defend against pathogens in *Schistosoma* (10). Both *Enterobacter* and *Klebsiella* found in the locust hindgut enhance host immunity through colony resistance (CR), reducing susceptibility to pathogenic infections (11). Imbalances in the composition and diversity of intestinal bacterial flora in *Spodoptera litura* larvae affect energy balance, metabolic homeostasis, and overall gut function (12). Reduced diversity of gut bacterial communities in wood pine larvae leads to slower growth, increased mortality, impaired pupation, and delayed adulthood (13). In summary, gut microorganisms play key roles in insect feeding, digestion, nutrition, immunity, and growth, ensuring proper physiological processes in their hosts.

Changes in insect gut microbial communities are influenced by environmental factors such as temperature, humidity, geography, and season, as well as the insects themselves, including their life history, physical and chemical gut environment, and gut structure (14–16). Host feeding habits result in significant differences in gut microbial communities, with laboratory-reared populations showing distinct microbial community distributions compared to field populations (17). In *Hermetia illucens*, Proteobacteria and Firmicutes dominate the midgut when fed a fish diet, while Proteobacteria dominates the midgut when fed a standard dipteran diet (18). Significant variations in gut microbial communities occur at different developmental stages, with microbial richness and diversity significantly higher in eggs than in adults throughout the life cycle of the caterpillar moth (17). The citrus fly exhibits significant changes in microbial species richness and dominant flora between eggs and larval adults, while the core flora remains relatively stable (19). Investigations on intestinal bacteria dynamics in black soldier fly indicate that the core microbiome, including *Citrobacter*, *Enterobacter*, and *Klebsiella*, dynamically changes with the metabolic demands of the host (20). Previous research conducted in the late 1950s accumulated numerous important observations on locust–bacteria interactions, some of which were summarized by Dillon and Charnley 20 years ago (21–23). They revealed that the most important bacterial phyla associated with the locust gut are Proteobacteria and Firmicutes (24, 25). Locusts undergo incomplete metamorphosis, transitioning through egg, larval, and adult stages, which inevitably leads to gut remodeling and potential changes in gut microflora. Research has shown that the core bacterial community of grasshoppers includes generally *Enterobacter*, *Klebsiella,* and *Pseudomonas*. However, whether the core gut microbiome undergoes significant changes and plays a crucial role in nutrient digestion in locusts remains uncertain. Locusts primarily feed on cellulose-rich plants containing polysaccharides such as cellulose, hemicellulose, lignin, and pectin (2). However, the complex multimeric structure of cellulose results in highly inefficient degradation through digestion. As a result, cellulose-feeding insects have evolved various survival strategies to release carbohydrates from resilient plant tissues, enabling them to overcome extreme nutritional barriers (26, 27).

Although previous studies suggest that gut bacteria play a significant role in digestion, absorption, and biomass conversion in locusts, direct evidence for their digestive function remains scarce. To address this issue, we investigated the gut microbiota structure of *L. migratoria* throughout their life cycle using high-throughput sequencing of 16S rRNA amplicons. We also assessed the digestive capacity of cellulose and hemicellulose at different life stages of locusts and isolated dominant gut bacteria under various conditions. We performed the DNS colorimetric method to study the cellulase activity of six bacteria *in vitro*. Subsequently, we identified key microorganisms responsible for nutrient digestion and growth in *L. migratoria*. This study aimed to provide crucial insights into industrial biorefinery processes for bioconversion of cellulosic feedstocks.

## MATERIALS AND METHODS

### Insect rearing and sample collection

Eggs of *L. migratoria* were harvested in Cangzhou, Hebei, China, and fed with wheat seedlings in the laboratory every morning from 8:00 to 9:00 am under controlled conditions of 28°C ± 1°C temperature, 60% ± 10% relative humidity, and a photoperiod of 14 (light):10 (dark), and the growth status of grasshoppers was observed.

*L. migratoria* (except eggs) were collected within 24 hours of moulting for each life stage (egg, first instars (L1), second instars (L2), third instars (L3), fourth instars (L4), fifth instars (L5), and imago), starved for 1 day, and dissected. Treatment of eggs was as follows: the eggs were digged in the soil, the pods were peeled off, and the egg grains were removed with a forceps. The soil attached to the surface was cleaned with water, followed by soaking in 75% alcohol for 1 minute to kill the surface bacteria, and finally the outside was cleaned with alcohol using sterile water, and all the above operations were carried out in a sterile operating table. Treatment of larvae was as follows: we used 75% ethanol to sterilize the larva for 1 min, and sterile water was used to wash the entire body to remove any microorganisms attached to the surface of the insects. *L. migratoria* L1, L2, L3, L4, and L5 were dissected to remove other tissues, leaving only digestive tissues, including the foregut, midgut, and hindgut. There were three biological replicates per group, and due to the small size of the eggs and L1, there were 15 flying locusts in each replicate group for the eggs and L1 and four grasshoppers in each of the remaining replicate groups. All samples were stored in a −80°C freezer.

### DNA extraction and PCR amplification

After homogenizing all the samples, DNA was extracted using the TGuide S96 Magnetic Soil/Fecal DNA Kit (Tiangen Biotechnology, Beijing, China). DNA quality was checked by gel electrophoresis on a 1.8% agarose gel and visually processed using a gel imager. The DNA concentration of the samples was determined using a Qubit dsDNA HS Assay Kit and a Qubit 4.0 fluorometer (Invitrogen, Thermo Fisher Scientific, Oregon, USA). The 16S rRNA was amplified using 27F and 1492R primers (27F: 5′-AGRGTTTGATYNTGGCTCAG-3′ and 1492R: 5‘-TASGGHTACCTTGTTASGACTT-3′) (28). For each sample, the 30-µL PCR mixture contained 1.5 µL of template DNA, 10.5 µL of enzyme-free sterile water (NFW), 15 µL of KOD ONE MM, and 1.5 µL of each upstream and downstream primer. The amplification products were tested for concentration (Qubit) and band (agarose gel electrophoresis), the qualified samples were mixed, and the final reaction products were purified using AMPure PB Beads (PacBio, USA) and placed on a Sequel II sequencer (PacBio, USA) for sequencing.

### High-throughput sequencing and analysis

The raw sequences obtained were filtered and demultiplexed using the SMRT Link software (v8.0) and minpass on raw reads generated by sequencing ≥5 and minPredictedAccuracy ≥0.9 to obtain circular consistent sequencing (CCS) reads. Quality filtering discards were performed by identifying forward and reverse primers using the Cutadapt quality control process (v2.7). The chimeric sequences were detected and removed using the UCHIME algorithm (v8.1) to obtain clean reads. The post-QC data were denoised using the DADA2 (29) method in QIIME2 (30) (version 2020.6), with a default threshold of 0.005% of the number of all sequences sequenced to filter ASVs. ASVs were classified based on a plain Bayesian classifier labeled QIIME2 (30) using the SILVA database [v132] (31) with a confidence threshold of 70%.

Alpha and beta diversities were calculated and displayed using QIIME2 to evaluate the degree of similarity between microbial communities in different samples. The observed Chao1, Shannon, and Simpson indices were used as indicators of alpha diversity, and principal coordinate analysis (PCoA), permutational multivariate analysis of variance (PERMANOVA), and nonmetric multidimensional scaling (NMDS) analysis were used to represent beta diversity. In addition, we used the effect size (32) to test for significant categorical differences between groups. A log LDA score of 4.0 was used as a threshold for discriminant features.

The taxonomic annotation of the feature sequences was carried out using SILVA as the reference database using a plain Bayesian classifier combined with the comparison method, and the taxonomic information corresponding to each feature was obtained. Then, the community composition of each sample was counted at each level (phylum, class, order, family, genus, and species), and the community structure was generated using the QIIME software. Different community structures of the samples at each taxonomic level were then mapped using R language tools. Prediction of the functional profiles of the bacterial communities based on 16S rRNA genes was achieved using a phylogenetic study (https://github.com/picrust/picrust2) of the communities by reconstructing unobserved states (PICRUSt2). Using the algorithm and software package PICRUSt, the functional class abundance of 16S rRNA gene sequences was predicted using the reference phylogeny to weigh the relative functional contribution of closely related sequences in the ASV table. Putative microbiota functions were derived as direct homologs of the Kyoto Encyclopedia of Genes and Genomes (KEGG). Metadata are also stored in the SRA (BioProject PRJNA990365); the serial numbers are SAMN36267132–SAMN36267152.

### Isolation, 16S rDNA sequencing, and physiological and biochemical analysis of *L. migratoria* intestinal bacteria

Five fourth and fifth instar larvae were randomly selected from plastic boxes. The larvae were sequentially sterilized with 3% ethanol and sterile water and dissected under sterile conditions. The intestines of *L. migratoria* were pooled in sterile Eppendorf tubes and homogenized with a sterile plastic pestle and mortar. Fresh homogenates of intestines were serially diluted to 10^−6^, 10^−8^, and 10^−10^ using sterile PBS. A total of 100 µL of each dilution suspension was dispersed in Luria–Bertani (LB) medium (10.0 g/L tryptic peptone, 5.0 g/L yeast, 10.0 g/L NaCl, and 15.0 g/L agar); *Lactobacillus* medium (De Man, Rogosa, and Sharpe[MRS]) (10.0 g/L casein digest, 10.0 g/L beef paste, 5.0 g/L glucose, 5.0 g/L sodium acetate, 5.0 g/L yeast powder, 2.0 g/L diammonium citrate, 1.0 g/L Tween 80, 2.0 g/L dipotassium hydrogen phosphate (K_2_HPO_4_-3H_2_O), 0.58 g/L magnesium sulfate (MgSO_4_-7H_2_O), 0.25 g/L manganese sulfate (MnSO_4_-H_2_O), and 18.0 g/L agar); and sodium carboxymethylcellulose medium (CMC medium) (2.5 g/L dipotassium hydrogen phosphate, 2.5 g/L disodium hydrogen phosphate, 20.0 g/L sodium carboxymethylcellulose, 2.0 g/L peptone, 0.5 g/L yeast dipping powder, and 14.0 g/L agar) and incubated at 30°C for 7 days. Colonies with different morphologies were isolated from each agar plate and transferred to the corresponding new agar plates for a second round of incubation. These colonies were analyzed according to their 16S rRNA genes. Universal bacterial primers 27F (5′-AGAGTTTGATCCTGGGCTCAG-3′) and 1492R (5′-TACGGYTACCTTGTTACGACTT-3′) (28) were used to amplify the 16S rDNA of the isolated strains. The PCR mixtures and amplification procedures were as described by Fang (33). Strains were classified using restriction analysis of amplified ribosomal DNA. PCR products were digested with AfaI and MspI. (TaKaRa, Kusatsu, Shiga, Japan) for 37 reactions per sample for 3 hours at 2°C,. DNA restriction endonuclease fragments were then separated by 2% agarose gel electrophoresis and visualized under UV light to identify the different ribotypes. Colonies with different ribotypes were assumed to represent different strains and were analyzed again for cultures 2 and 3. A strain was considered purified if the restriction analysis pattern of amplified ribosomal DNA was the same for all three transfer cultures of a colony. The 16S rRNA genes of all purified isolates were sequenced by Bioengineering Biotechnology Ltd (Shanghai, China) using Sanger sequencing. Sequences were assembled using SeqMan software and compared with reference sequences in the GenBank database (http://www.ncbi.nlm.nih.gov/BLAST). All purified strains were maintained in the corresponding medium supplemented with 20% glycerol at −80°C for further use. Physiological and biochemical characterization of the six bacteria was performed at 6°C using API ZYM and 1 CH kits (bioMérieux, Marcy-l'Étoile, France), respectively, according to the manufacturer’s instructions (34).

### Cellulose and hemicellulose digestibility

Laboratory-fed grasshoppers were grouped according to instar, with *L. migratoria* divided into six groups (L1, L2, L3, L4, L5, and imago). Grasshoppers were fed fresh wheat seedlings (*Triticum aestivum* Linnaeus, 1753) grown in the laboratory, and the specific feeding procedure, preparation of the fecal sample solution, and wheat seedling sample solution were performed as described by Wang (35). The dry-to-fresh ratio of the wheat seedlings was determined as stipulated by Subrata et al. (36).

Glucose and xylose standard curves were plotted with reference 37. The linear equation of glucose obtained in this experiment was *y* = 1.7321*x* + 0.0083, *R*^2^ = 0.9955, and the linear equation for xylose was *y* = 53.96 *x* − 0.0044, *R*^2^ = 0.9969. “*x*” is the reducing sugar concentration, and “*y*” is the absorbance value.

The absorbance values of the reaction solutions of fecal and wheat seedling samples were determined using the anthrone and moss black phenol colorimetric methods, respectively, as per Wang et al. (35). The cellulose and hemicellulose digestibility of grasshoppers on wheat seedlings was calculated using the following equations:


$$\begin{array}{c} Cellulose\;(hemicellulose)\;content\;(\%)=\frac{c\;\times \;240\;\times \;10^{-3}\;L\;\times \text{ dilution multiple }\times \;0.9(0.88)}{m}\times 100\%\\ Cellulose\;\left(hemicellulose\right)\;digestibility\;\left(\%\right)=\frac{a\;-\;b}{a}\times 100\% \end{array}$$

Note: *c* is the sugar concentration (g/L) of the feces calculated according to the standard curve, 240 is the total volume of the sample solution (mL), m is the weighed sample mass (g). 0.9 and 0.88 are coefficients, *a* is the amount of cellulose fed on wheat seedlings (g), and *b* is fecal cellulose content (g).

DNS colorimetric as well as qualitative filter paper methods were used to determine cellulase activity as well as filter paper activity. Three parallel experiments were set up for each group, and a three-tube control experiment was performed. Referring to reference 37, the optical density (OD) values measured for each experimental group were brought to the glucose standard curve to obtain the glucose concentration, which in turn was used to calculate the cellulase activity value of each group using the following formula.


$$\text{ enzyme activity }(U/mL)=\frac{A\times n\times 1,000}{t\times v}$$

Note: *A* is the glucose content (mg) obtained by substituting the measured OD value into the glucose standard curve, *n* is the number of dilutions, 1,000 is the unit mg converted to μg, *t* is the color development time, and *v* is the volume of the crude enzyme liquid.

### Statistical analysis

Data obtained from different groups were evaluated using analysis of variance (ANOVA), and multiple comparisons tests were performed using the Student–Newman–Keuls test. All analyses were performed using GraphPad Prism9. The level of significance was set at *P* < 0.05 or *P* < 0.01 (38). The top 10 genera of relative abundance in the intestines of *L. migratoria* of different ages were subjected to Spearman’s correlation analysis (γ = 0.1; *P* = 0.05) with cellulose digestibility and hemicellulose digestibility, and correlation heat maps were drawn. This analysis was performed on Biomarker Cloud Platform (Biomarker Biotechnology Co.)

## RESULTS

### Diversity of bacterial communities throughout the life cycle of *L. migratoria*

**Fig 1:**
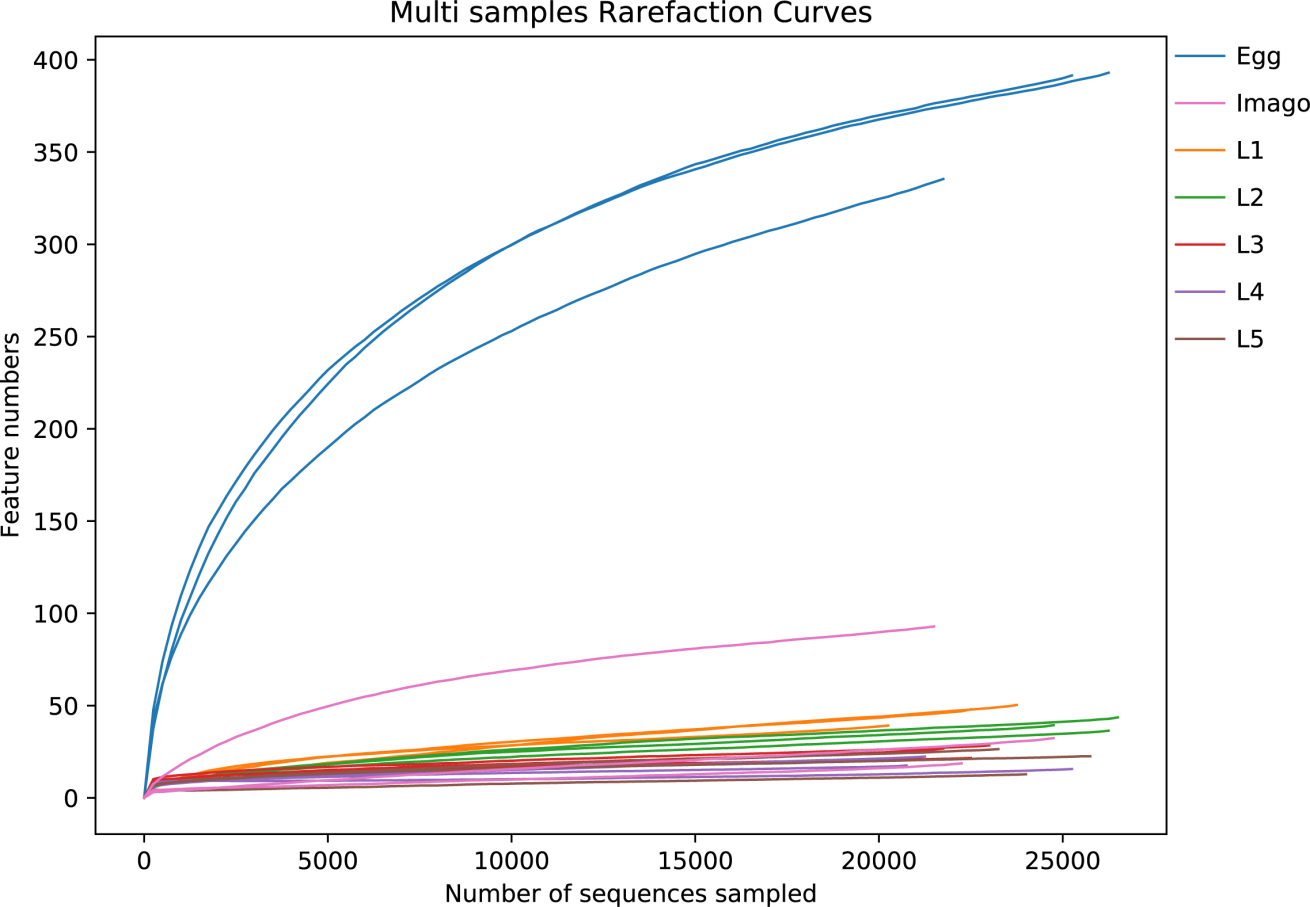
Rarefaction curves. Dilution curve to verify whether the amount of sequencing data is sufficient to reflect species diversity and indirectly the species richness in the sample. The horizontal axis is the number of randomly selected sequences, and the vertical axis is the number of features obtained based on the number of sequencing entries; each curve represents one sample.

The negative control (NC) sample showed an obviously different bacteria community with fewer shared ASVs than those in treatment groups, confirming the absence of contaminants in our experiment. Thus, the NC was excluded in the following analysis. A total of 515,478 raw sequences were obtained by full-length high-throughput sequencing of 16S rDNA from 21 samples of *L. migratoria* at different life stages. After length filtering and removal of chimeras, the final sample size contained 503,522 sequences (species richness ≥2), which were clustered at 100% sequence similarity to generate a total of 600 ASVs. Figure 1 shows that the rarefaction curve of all samples leveled off and allowed for data analysis. In total, 20 phyla, 33 orders, 171 families, 302 genera, and 477 species were identified.

**Fig 2:**
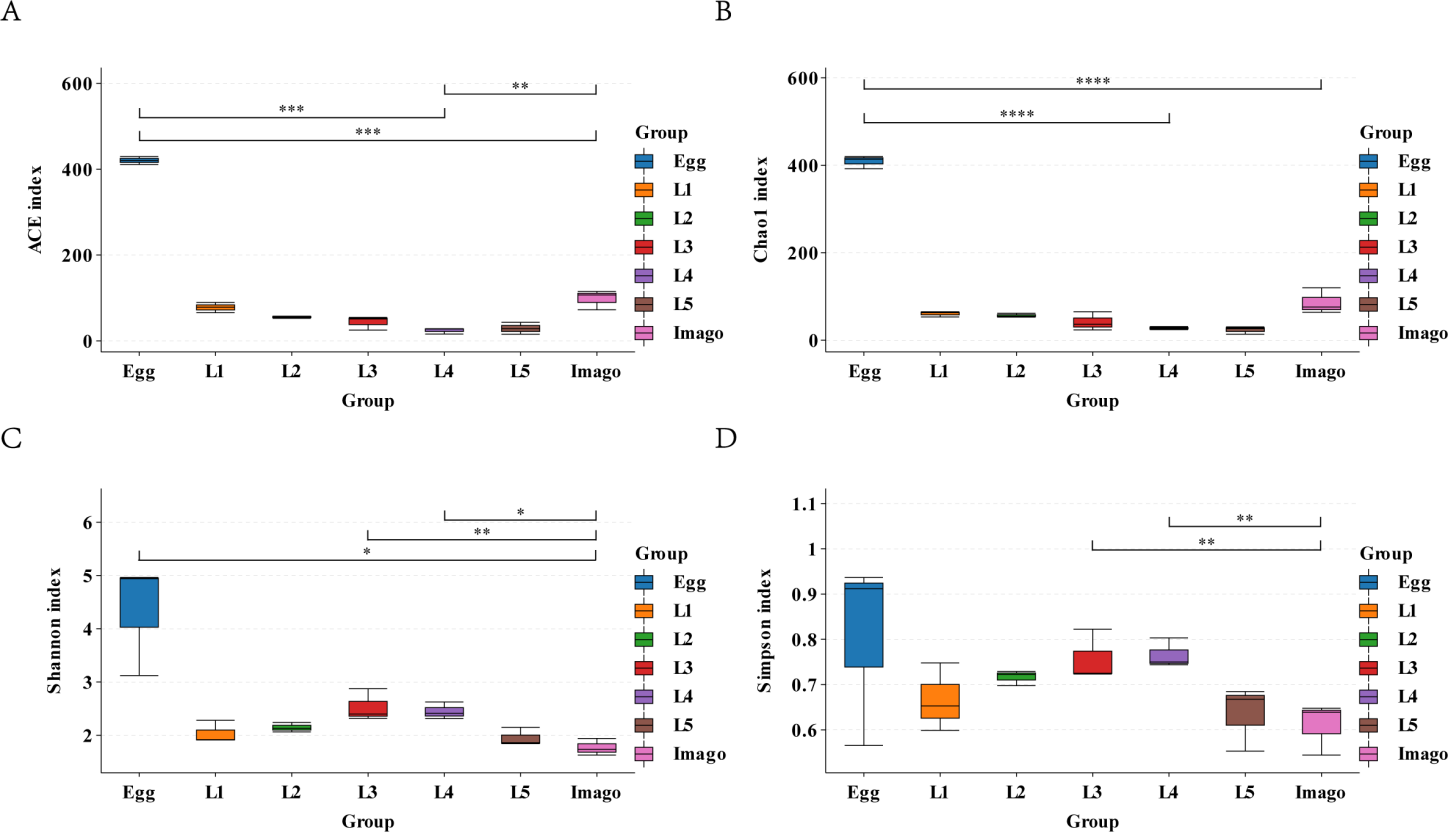
Alpha diversity analysis. (**A**) ACE index plot, (**B**) Chao1 index plot, (**C**) Shannon index plot, and (**D**) Simpson index plot. The horizontal axis represents the different life stages, and the vertical axis is the corresponding alpha diversity index values. In box line plots, upper and lower end lines are the upper and lower quartiles, respectively; the median line is the median; the upper and lower edges are the maximum and minimum inner circumference values, respectively. * and ** represent significant differences; *** and **** represent highly significant differences.

The alpha diversity of each *L. migratoria* life stage was analyzed based on the OUT table using the ACE, Chao1, Shannon, and Simpson indices (Fig. 2). Based on the Chao1 and ACE indices (Fig. 2A and B), species richness was significantly higher in eggs than in larvae and imagoes (Student’s *t*-test, *P* < 0.01); in larvae, species richness was significantly higher in L1 and L2 than in L3 (Student’s *t*-test, *P* < 0.05). Species richness was significantly higher than in imagoes than in fourth and fifth instar larvae, but still significantly lower in L1.

The Shannon index ranged from 1.6281 to 4.9607, and the Simpson index ranged from 0.5442 to 0.9368 for the life stages of grasshoppers (Fig. 2C and D). The species diversity of eggs was significantly higher than that of imagoes based on the two indices, with significant differences between imagoes and L3, L4, and L5, but no significant differences between the remaining life stages. Species diversity was higher in L3 and L4 based on the Shannon index. In addition, the species distribution of eggs was extremely heterogeneous throughout the life stages, and the species distribution was the most uniform in L2.

**Fig 3:**
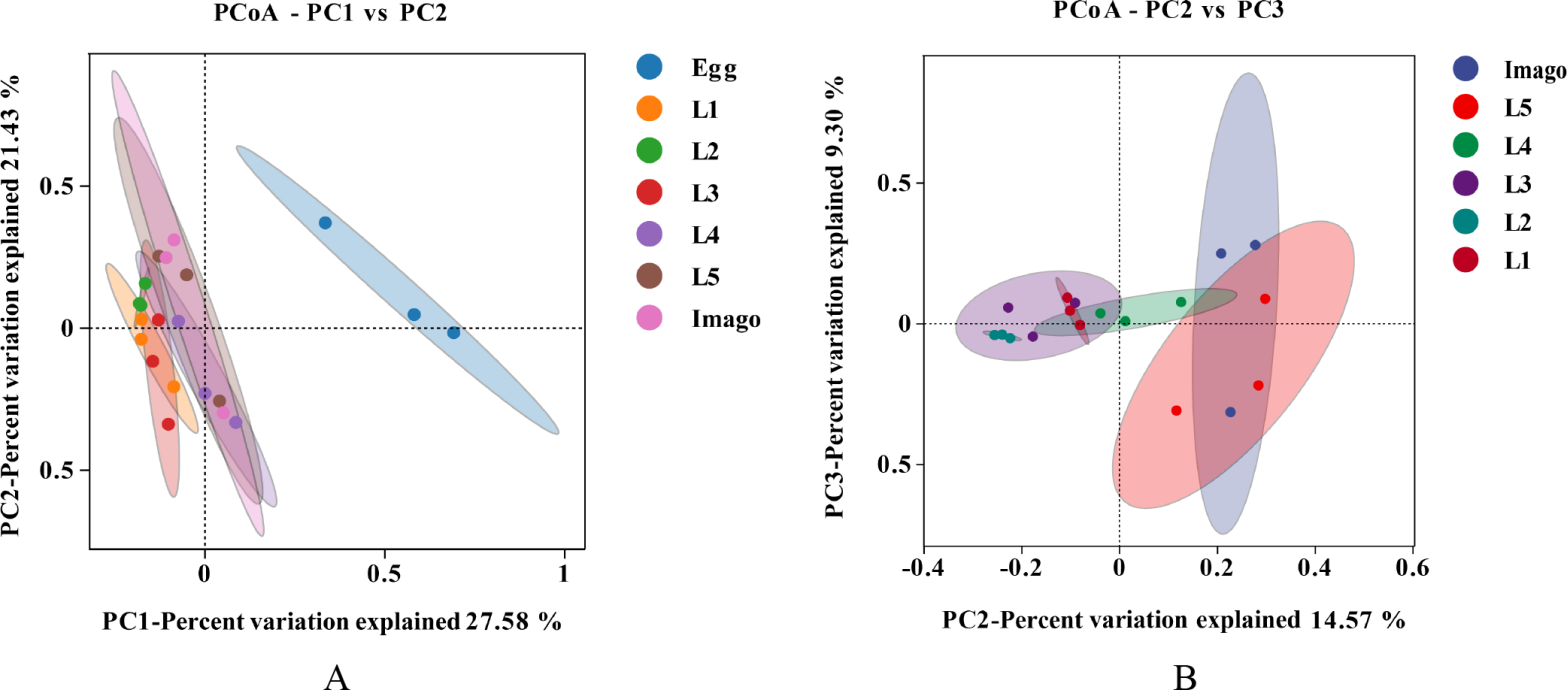
Principal component analysis. (**A**) Egg to imago stage. (**B**) L1 to imago stage. Each point represents a sample, and each color represents a sample group (if any); ovals enclose 95% confidence ellipses. The horizontal and vertical axes indicate the first (PC1) and second (PC2) principal components, respectively. Percentages indicate the contributions to sample variance.

Principal coordinate analysis (PCoA; Fig. 3) based on the Bray–Curtis algorithm also demonstrated differences in gut microorganisms among samples of different ages, indicating that the gut bacterial community changes dynamically throughout the growth and development of *L. migratoria*. PERMANOVA allowed us to test whether beta diversity was significantly different between samples of different subgroups. The significance of this differential separation was further confirmed by PERMANOVA (*R*^2^ = 0.577, *P* = 0.001), indicating a high confidence in the principal coordinate analysis (PCoA) test. PCoA showed that the larvae and adults were distributed away from the egg, indicating that eggs had a more distinctive microbiota composition. The distances of L3, L4, and L5 from imagoes were lesser than those of L1 and L2, indicating that the microbial diversities of L3, L4, and L5 were closer to that of the imago. In addition, the previously observed heterogeneity of eggs with L2 was supported by the PCoA.

### Species composition of *L. migratoria* at different developmental stages and core bacterial community

**Fig 4:**
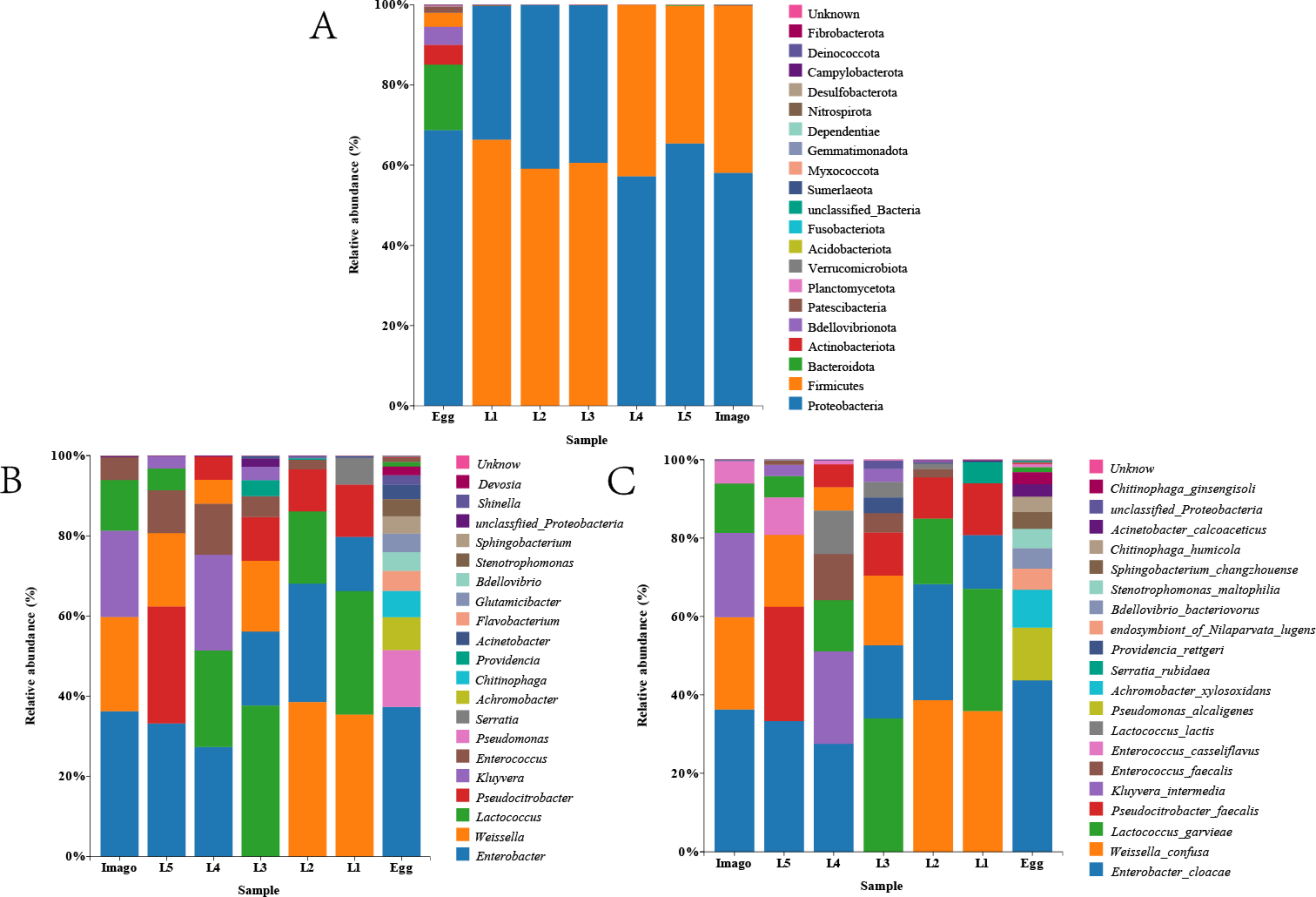
Species composition histograms. (**A**) Distribution at the phylum level, (**B**) distribution at the genus level, and (**C**) distribution at the species level. Each plot contains the top 20 dominant bacteria. The color in the lower left represents the color of the group in which the sample was located; the color in the upper right represents the top 20 species ranked by table species richness. Species without annotations are classified as “unclassified.”

Taxonomic analysis (Fig. 4) at the phylum level showed that the eggs were richer in bacterial species; other periods had fewer species. Proteobacteria, Firmicutes, Bacteroidetes, and Actinobacteria dominated all the bacterial communities at the phylum level. Firmicutes was the first dominant group in L1, L2, and L3, accounting for 66%, 59%, and 60%, respectively. In L4, L5, and imago, Firmicutes dropped to the second position, accounting for 42%, 40%, and 41%, respectively; however, Firmicutes abundance was only 3% in eggs. Bacteroidetes ranked in the top three in eggs (16.8%), but was barely detected in the other biological samples (< 1%). At the genus level, *Enterobacter* had the highest percentage of all biological samples (13.5%–35.3%). *Weissella* ranked in the top three at L1, L2, L3, L5, and imago of *L. migratoria*, but dropped to the fifth or sixth position at L4. *Lactococcus* was the dominant genus in *L. migratoria*, but the percentages of this organism at other stages were significantly higher than that at L5. *Pseudocitrobacter* was the dominant genus in all stages, except imago, with the highest percentage of 28% in L5 and only 0.2% in eggs. *Kluyvera* was only present in L3, L4, L5, and imago and had a higher proportion in L4 and imago (24.6% and 21.8%, respectively). *Serratia* was only present in L1 and was barely detectable in other biological samples (< 1%). Similarly, *Providencia* was only present in L3 samples and barely detected in other samples (< 1%). In the samples we studied, such as *Enterococcus*, the percentage of bacterial abundance fluctuated between samples of different ages, with a maximum of 12.6% in L4 and a lower percentage in eggs (0.7%). A similar phenomenon was observed at the taxonomic level in this species. For example, *Enterobacter cloacae* was present in all microbial communities at high abundance, and its content varied between samples of different stages, with 35.3% in imago, 18.3% in L3, and 13.5% in L1. *Weisseria* occupied a major position throughout the larval and imago periods, with the highest proportions in L1 and L2 (33.9% and 38.2%, respectively). *Lactococcus*, *Pseudococcus*, *Kluyvera intermedia*, and *Enterococcus faecalis* were detected in nearly all samples. *Serratia rubidaea* was only present in L1 and eggs, and *Providencia* was only present in L3 and L2.

**Fig 5:**
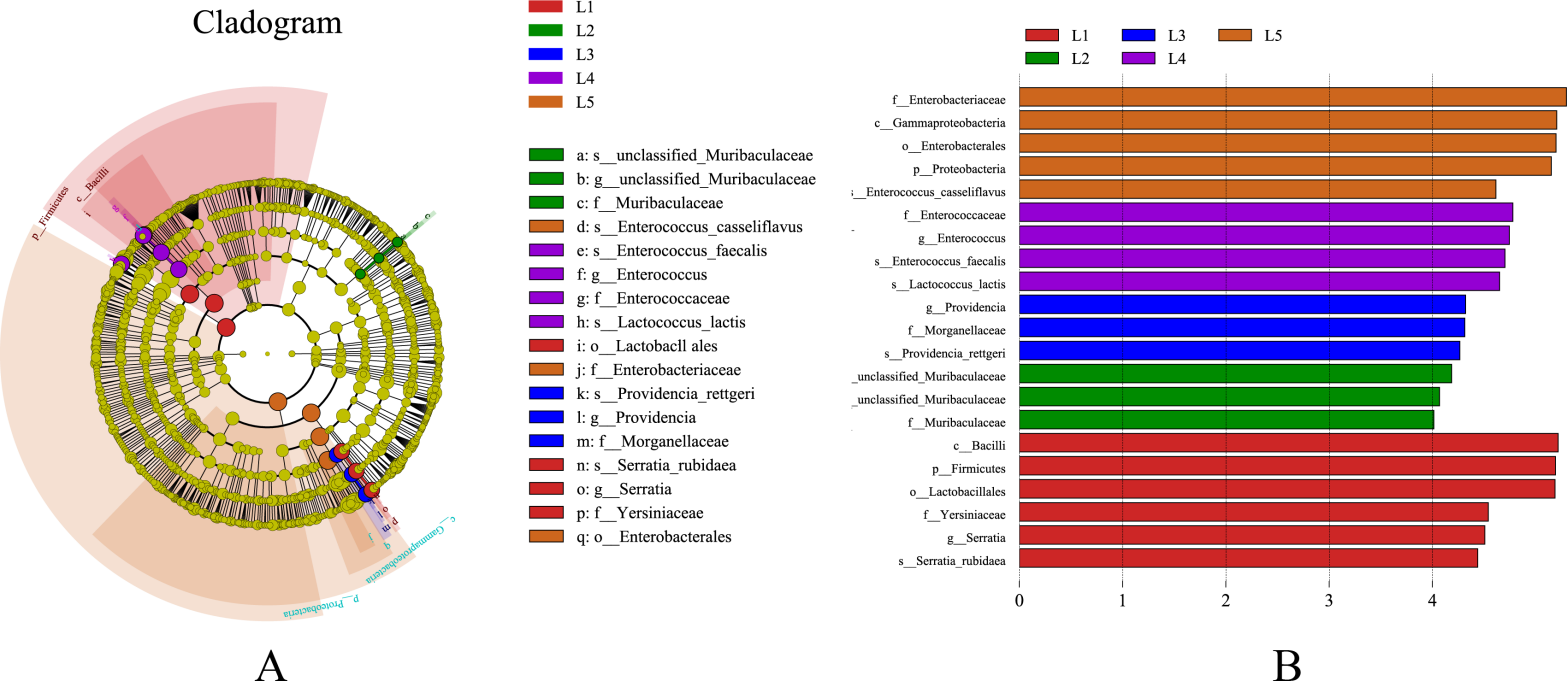
Linear discriminant analysis effect size (LEfSe) analysis diagram. (**A**) Circles radiating from the inside to the outside of the evolutionary branching diagram represent the taxonomic levels from phylum to species; each small circle at a different taxonomic level represents a taxon on at that level, and the diameter of the small circles is proportional to the relative abundance; yellow indicates no significant differences; other differences are colored according to the group with the highest abundance of the species. Different colors indicate different subgroups, and different colored nodes indicate the microbiota that play an important role in the subgroup. (**B**) The vertical axis shows the taxonomic units with significant differences between groups; the horizontal axis shows a bar graph visualizing the logarithmic score of LDA for each taxonomic unit. Taxa are sorted according to their scores, with longer lengths indicating more significant differences between taxa. The color of the bars indicates the sample group with high abundance for that taxon.

Linear discriminant analysis effect size (LEfSe) analysis (Fig. 5) was employed to identify specific bacterial taxa responsible for significant variability across different life stages of *L. migratoria*, ranging from the phylum to genus level, with a threshold of LDA scores higher than 4.0. Evolutionary branching maps were constructed to provide a comprehensive understanding of the taxonomic distribution and evolutionary competence of microbial communities throughout the life stages. However, adults were excluded from the analysis as they did not exhibit any specific bacterial taxa (LDA scores below 4.0), causing significant variation. In the first instar larvae, four specific microbial taxa were identified, belonging to the genera *Serratia*, *Lactococcus*, and *Bacillus*. Similarly, the second instar larvae showed three specific microbial taxa, attributed to the genus *Mucispirillum*. For the third instar larvae, three specific microbial taxa were observed, associated with *Providencia* and *Morganella*. The fourth instar larvae exhibited four specific microbial taxa, belonging to *Enterococcus* and *Lactococcus*. Lastly, the fifth instar larvae screened positive for five specific microbial taxa, including *Enterococcus*, *Enterobacter,* and *Pseudomonas*.

**Fig 6:**
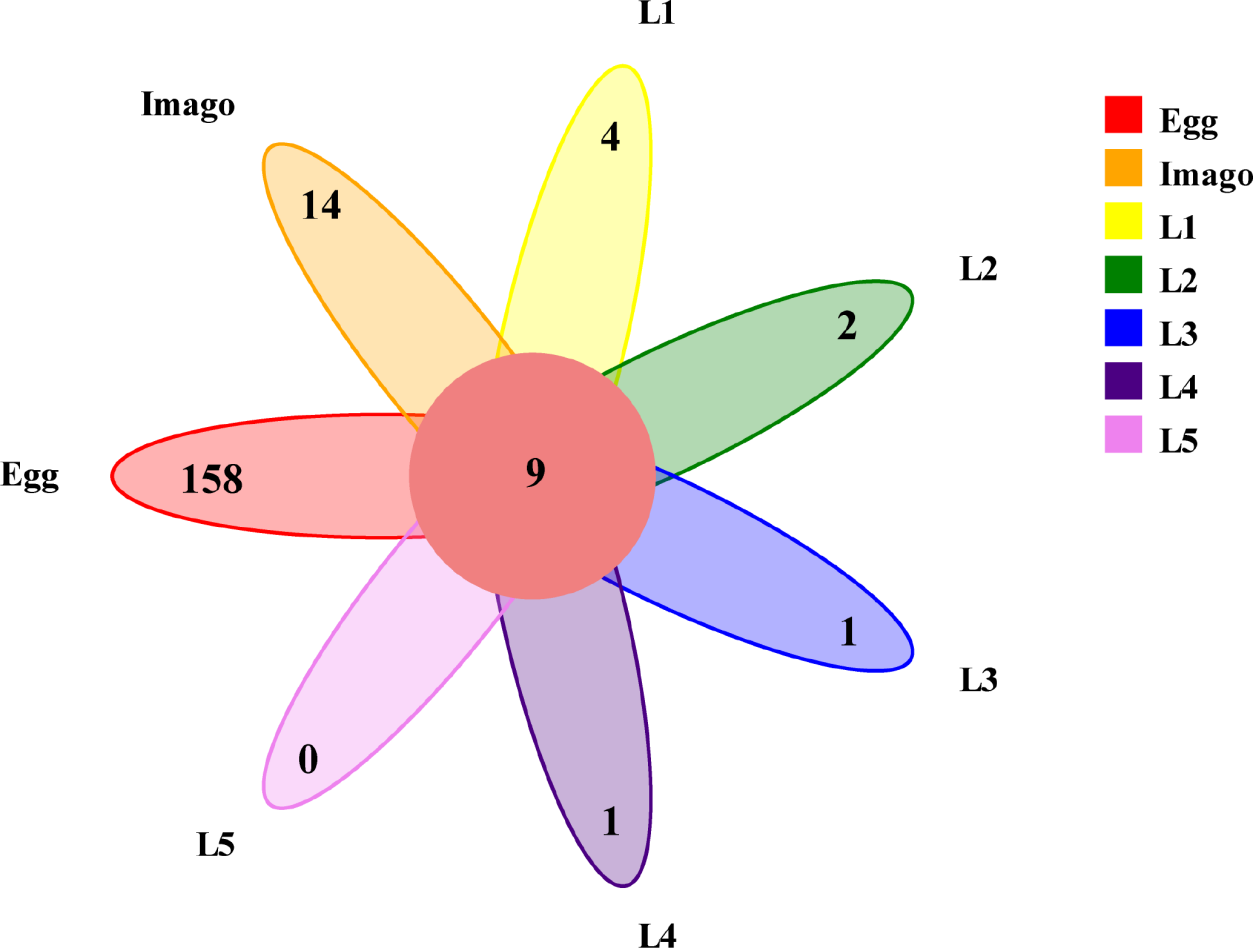
Venn diagram (genus level unit). Colors represent different samples/groups, overlapping parts represent the number of features common to the samples/groups, and non-overlapping parts represent the number of features specific to the samples/groups.

To determine the core gut microbiota, we used a Venn diagram to include the genera present in all samples as criteria for the core. As shown in (Fig. 6), the core of the microbiota consisted of nine bacterial genera with 11 ASVs, and the genera that accounted for the highest proportions were *Enterobacter*, *Lactococcus*, *Pseudocitrobacter*, and *Enterococcus*. We speculate that the reason for the abundance of these microbes in grasshoppers is that these microbes are good opportunistic colonizers. The co-occurrence of these genera suggests that they play an important role in the growth and development of *L. migratoria*.

### Functional prediction of intestinal bacteria in *L. migratoria*

**Fig 7:**
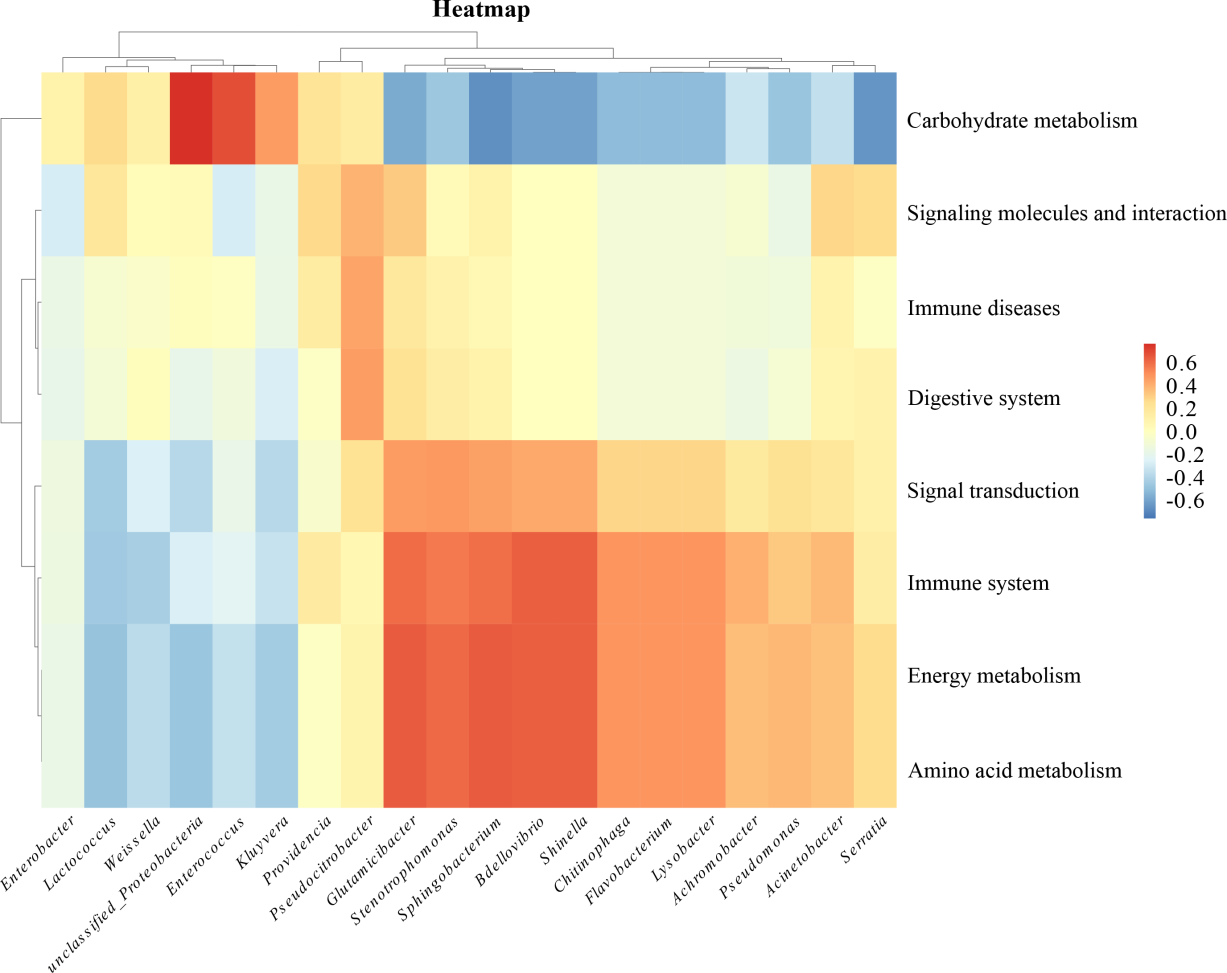
Functional prediction heat map. The horizontal axis indicates intestinal bacteria; the vertical axis indicates related functions. Red indicates a positive correlation, blue indicates a negative correlation, and the shade of color indicates the degree of correlation.

To better understand the functionality of the potential gut microbiome, we used the Picrust2 software to correlate the 16S rRNA profiles of each sample with the genomically derived functional pathways from bacterial sequencing (Fig. 7). We selected pathways with relative abundances of >1%, including environmental information processing (membrane transport), genetic information processing (replication and repair), and metabolism (carbohydrate, vitamin, lipid, amino acid, and nucleotide metabolism). These pathways have been validated in KEGG (https://www.kegg.jp/). The aforementioned analysis showed that *Proteobacteria* and *Enterococcus* were significantly enriched in carbohydrate metabolism *Glutamicibacter, Sphingobacterium,* and *Bdellovibrio* showed a negative correlation in carbohydrate metabolism; and *Glutamicibacter, Sphingobacterium*, *Bdellovibrio,* and *Shinella* were significantly enriched in amino acid metabolism pathways as well as in the immune system. The prediction of bacterial function can help us screen target strains with purpose and direction.

### Bacteria isolated from the intestines of *L. migratoria*

To study the physiological functions of intestinal microorganisms, bacteria were isolated from the intestines of *L. migratoria* under different conditions. A total of three bacterial strains were isolated on the CMC medium and identified as related to Proteobacteria based on the phylogenetic analysis of 16S rRNA genes. A total of five bacterial strains were isolated on the LB medium, belonging to the Proteobacteria and Firmicutes. A total of five bacterial strains were isolated on the MRS medium, belonging to *Clostridium*, *Fusobacteria*, Proteobacteria, and Firmicutes. Thirteen strains were re-inoculated onto the LB medium, and the duplicate strains were filtered. Eight strains were then screened, and six different genera were selected after Sanger sequencing and comparative analysis of the eight strains. Strains LM1, LM2, LM3, LM4, LM5, and LM6 belonged to the genera of *Pseudomonas*, *Lactococcus*, *Clostridium*, *Enterobacter, Bacillus*, and *Serratia*, respectively, and the cellulose and filter paper enzyme activities of these six strains are shown in Table 1.

**TABLE 1:** Enzyme activities of isolated strains.

Strain	Enzyme activity (U/mL)	
	CMCA	FPA
LM1	5.766	4.096
LM2	0.358	1.0739
LM3	1.031	1.1697
LM4	0.5076	0.7238
LM5	3.459	4.737
LM6	2.987	3.529

### Cellulose and hemicellulose digestibility in the intestines of *L. migratoria* of different stages

**Fig 8:**
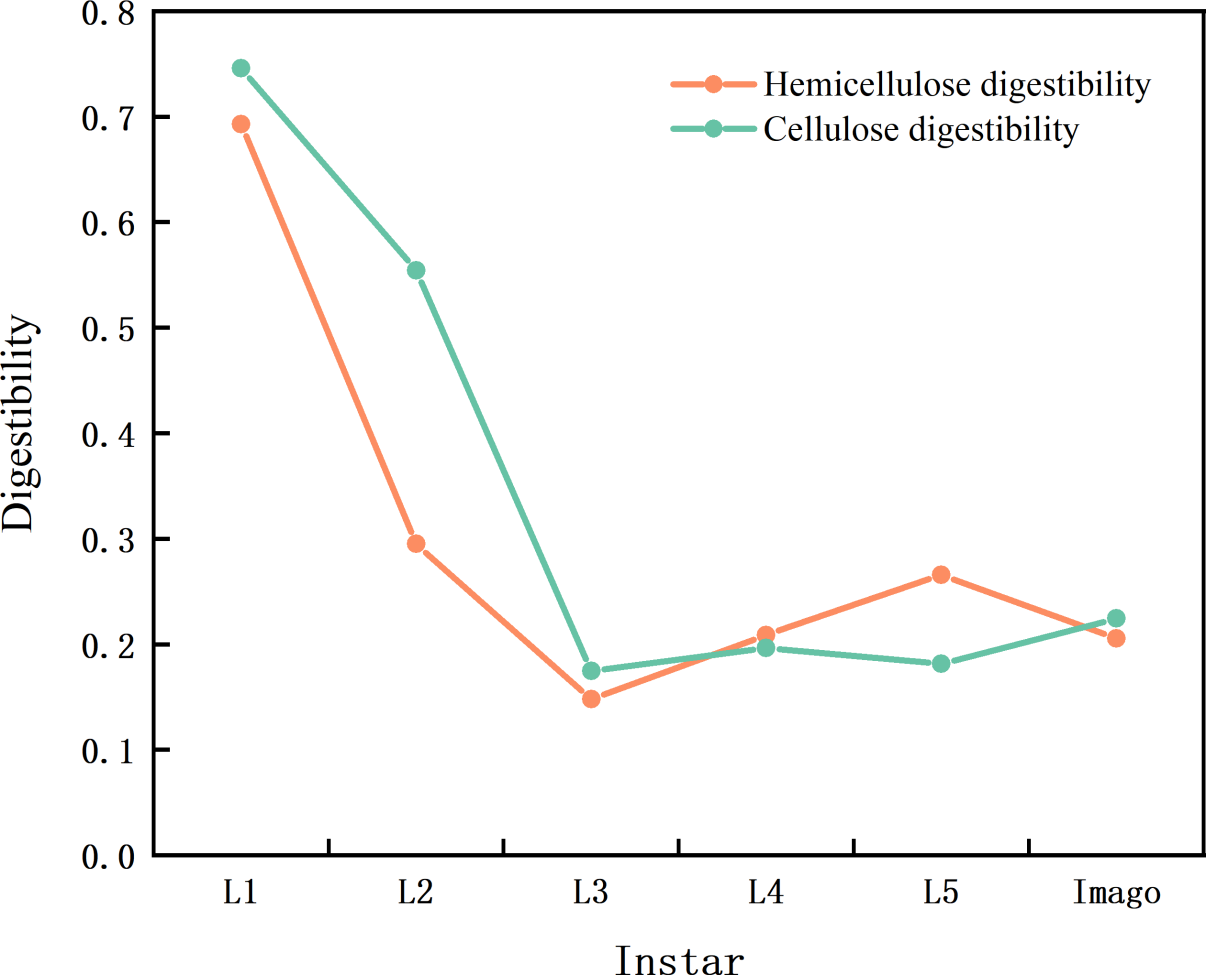
Cellulose and hemicellulose digestibility. The orange curve is hemicellulose digestibility, the green curve is cellulose digestibility; the abscissa is instar, and the ordinate is digestibility.

To understand the effect of these bacteria on the growth and development of locusts, we analyzed the cellulose and hemicellulose digestibility of locusts at different stages. The results are displayed in Fig. 8. The results showed that cellulose and hemicellulose digestibility had the same trend, which was highest at the first stage and stabilised after the third stage. This trend is similar to the changes in gut bacterial diversity at different stages. The early microbiota is likely to contain gut microorganisms from the environment, and as the *L. migratoria* matures, these strains may be replaced by more stable resident bacteria, similar to the different stages found in that of human infants (39).

### Correlation between gut microorganisms and cellulose and hemicellulose digestibility in the *L. migratoria*

**Fig 9:**
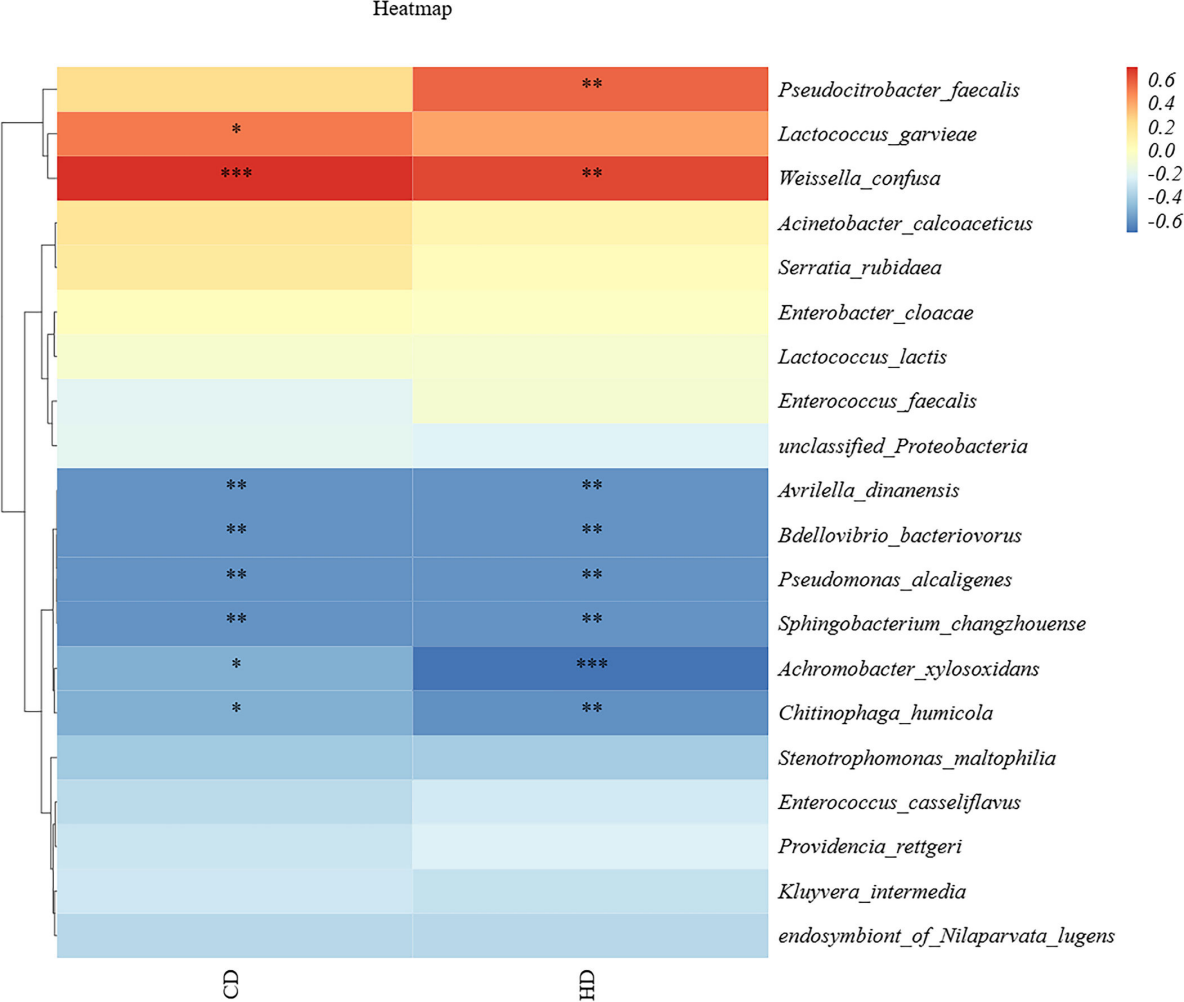
Correlation of cellulose and hemicellulose digestibility. The abscissa is digestibility, HD is hemicellulose digestibility, CD is cellulose digestibility, and the ordinate is the dominant bacteria common to all ages; darker colors indicate a greater correlation; red indicates a positive correlation, and blue indicates a negative correlation. A number of “*” above 2 is highly significant correlation. A “*” indicates significant correlation.

To further understand the relationship between locust gut microorganisms and cellulose digestion, a correlation analysis was performed between cellulose and hemicellulose digestibility and the dominant strains in the gut. We selected the top 20 dominant strains in terms of abundance shared across the life stages and analyzed them in conjunction with the determined cellulose and hemicellulose digestibilities, obtaining the results displayed in Fig. 9. As can be seen in the Fig. 9, *Weissella confusa, Pseudocitrobacter faecalis*, and *Lactococcus garvieae* were significantly positively correlated with cellulose and hemicellulose digestibility. Meanwhile, *Avrilella dinanensis* and *Bdellovibrio bacteriovorus* were negatively correlated with cellulose digestibility, and *Achromobacter xylosoxidans* and *Bdellovibrio bacteriovorus* were negatively correlated with hemicellulose digestibility. Interestingly, *Weissella*, *Enterobacter*, and *Pseudocitrobacter* were the dominant genera in the locust gut.

Species-level distribution maps indicate that *Enterobacter cloacae*, *Pseudomonas aeruginosa*, and *Lactococcus lactis* were dominant in almost all larval ages. *Clostridium lyticum*, *Bacillus*, and *Escherichia hermannii* were not highly abundant in different larval ages, but were frequently isolated from the intestines of *L. migratoria* and other insects (40–42). Cellulase and filter paper activities of strains LM1–LM6 were determined using the DNS colorimetric method. Cellulase is a complex enzyme that includes endoglucanase, exoglucanase, and β-glucosidase, and the CMC enzyme activity represents the activity of endoglucanase, while the FPA enzyme activity is used to represent the total enzyme activity of the aforementioned three enzymes (43). Results showed that LM1 and LM6 had a higher cellulase activity than LM2 and LM4, while LM1 and LM5 had a higher filter paper activity than LM4. These enzymes included β-galactosidase, α-glucosidase, and β-glucosidase. Comparison of cellulose and hemicellulose utilization and enzyme activities of these six bacterial species showed that their physiological properties differed considerably.

## DISCUSSION

In this study, we performed high-throughput sequencing of the 16SrRNA gene to examine the diversity and community structure of gut bacteria throughout the life cycle of *L. migratoria*. This is the first time that the gut microbes of *L. migratoria* at different life stages have been dynamically monitored under laboratory conditions (including egg, L1, L2, L3, L4, L5, and imago). Gut microbial diversity exhibited dynamic changes throughout the life cycle of *L. migratoria*. Specifically, the egg stage displayed significantly higher gut microbial diversity than the adult stage; also, the first and second instar larvae showed higher diversity than the third instar larvae. Nevertheless, the taxonomic composition of bacteria demonstrated a relative stability at higher taxa. This finding aligns with similar observations in other insects, including honeybees (44), dragonflies (45), beetles (46), and leafhoppers (47). Moreover, beta diversity results illustrated that the gut microbial community exhibited greater variability in early larvae in contrast to late larvae and adults, which supports the theory of microbial community composition (48). Considering the ongoing development of the immune and digestive systems in early larvae (39), it is plausible that their microbiota originates from the environment, and as *L. migratoria* mature, these strains may be replaced by more stable resident bacteria. This phenomenon resembles the stages observed during the establishment of the human infant microbiota (49). Evidence suggests that the association between ecological niche effects and gut microbiota in the field is dependent on the developmental stage of the insect, which is consistent with our findings. This further suggests that grasshoppers can predict changes in their gut microbiota throughout their life history.

Our findings indicate that the majority of bacteria found in *L. migratoria* larvae and imago can also be detected in eggs, suggesting a possible vertical transmission of gut microorganisms to the offspring. It has recently been proposed that *Klebsiella* and *Enterobacter*, which are associated with the locust gut, use the egg pod as a medium for transmission (50). This transmission process plays a vital role in providing essential nutrients such as vitamins, nitrogen, and amino acids that are deficient in the diet, thereby facilitating effective transmission between populations. Interestingly, despite the limited microbiota present in the eggs, *Enterococcus* successfully occupies an ecological niche, establishing stable colonization and transmission to the larval gut, a phenomenon observed in locusts. The reason for this phenomenon, which is different from the stabilization of colonization achieved by microorganisms by reducing their own abundance to reduce potential competition, remains to be explored in depth. However, besides maternal transmission, it should be noted that some bacteria might also be acquired from external sources such as food or group contact during the growth and development of *L. migratoria*. In support of the food origin hypothesis, Vaughan et al. (51) analyzed the dominant genera of endophytic microorganisms in the berry borer gut, providing strong evidence for the relationship between gut microorganisms and their food source (berries).

This study found that Firmicutes and Proteobacteria dominated all life stages in the gut of *L. migratoria*, similar to previous studies on other adult grasshoppers under field conditions, including *Sphingonotus mongolicus* (52), *Oedaleus decorus* (53), and *Schistocerca gregaria* (54). Proteobacteria and Firmicutes are also the main phyla in other insects, including praying mantis, *Anopheles bicolor*; rainbow stag beetle, *Holotrichia parallela*; and burying beetle (35, 55–59). In addition, the present study found that the age of *L. migratoria* was negatively correlated with the Proteobacteria phylum and positively correlated with Firmicutes, which is related to the function of the anamorph phylum in inhibiting the aggregation of *L. migratoria*. Previous studies demonstrated that the intestinal Firmicutes plays a crucial role in degrading complex plant carbohydrates, thereby enhancing the food digestion capacity of the host (60). The diversity profiles of gut bacteria in *L. migratoria* indicate the highest diversity in eggs, which decreases gradually from the first to the third instar and stabilizes thereafter until adulthood. This trend corresponds to the digestibility of cellulose and hemicellulose that we measured, implying that bacterial diversity influences their digestibility in *L. migratoria*. The consistent presence of *Enterobacter* bacteria in the hindgut has been repeatedly confirmed, indicating a selective interaction between locusts and these bacteria (48, 61, 62). Shi et al. (63) reported that an entomopathogenic fungus almost entirely inhibited the growth of isolated *Enterobacte*r bacteria in the hindgut by lowering the pH from 6.3 to 5.6, suggesting that the hindgut is characteried by specific conditions conducive to the growth of certain intestinal bacteria, thus further confirming their adaptive growth in the gut and the close symbiotic relationship between locusts and specific bacterial species, such as species of *Enterobacter*. Such a relationship is closely linked to the function of *Enterobacter*. Inoculation of *Enterobacter* increased egg weight and male fitness, leading to an improved insect fitness through additional bacterial implantation (64). Sami et al. (65) discovered that *Enterobacter* can produce endo-1,4-glucanase, which aids insects in cellulose digestion. Correlation analysis in our study reveals a significant positive correlation between *Lactococcus*, *Weissella*, *Pseudomonas* and the digestibility of cellulose and hemicellulose, suggesting that these bacterial phyla enhance the cellulose digestion ability of the host. Our study also finds a high proportion of *Weissella* in *L. migratoria* gut microbiota, consistent with the report of Tang et al. (66) wherein *Weissella* is strongly associated with swarming locusts. Moreover, Wada-Katsumata (67) showed that *Weissella* is widespread in swarming cockroaches, where it causes aggregation of German cockroaches, further implying that *Weissella* contributes to swarm formation and insect adaptation to their environment. Although species abundance plots indicate that *Weissella* is the dominant genus, yet we did not isolate it in this study because it is an anerobic bacterium, which makes it difficult to culture.

*In vitro* enzyme activity measurements were conducted using the screened isolates to assess the cellulase activity of dominant strains in *L. migratoria*. The results underscored that different bacteria exhibit significantly different enzyme activities for cellulose, indicating that gut microorganisms possess the enzymatic capability required to degrade cellulose and hemicellulose. A previous study has demonstrated that *Bacillus* isolated from the intestinal tract of the desert locust can decompose cellulose due to the presence of a gene encoding cellulase (68). Additionally, three strains of cellulolytic bacteria (*Paenibacillus*, *Bacillus*, and *Aeromonas*) were isolated from the sugar industry waste stream (44). *Bacillus* exhibits strong protease, amylase, and lipase activities, as well as enzymes for degrading plant complex carbohydrates such as cellulase and dextranase. These enzymes are capable of destroying the cell wall of the plant cell, promoting the release of nutrients and eliminating antinutritional factors in plants. In this study, we also isolated *Bacillus*. After CMC and FPA determination, it was shown to have strong cellulase activity as well, in agreement with previous studies. A strain of *Aeromonas* isolated from soil was found to have cellulolytic ability (69), and another study discovered that *Klebsiella* isolated from the hindgut of crickets also exhibit cellulolytic ability (70). Determination of the cellulase activity of cellulolytic bacteria in the intestines of Siberian locusts from Xinjiang grasslands using the DNS method indicated that the cellulase activity ranges from 0.84 U/mL to 1.63 U/mL for several bacterial strains isolated (71), which is similar to LM2, LM3, and LM4. LM1 and LM5 recorded the highest cellulase activity and filter paper activity in this study, which was three times higher than that in the other three strains, suggesting that *Pseudomonas* and *Enterobacter* have the capacity to degrade cellulose efficiently.

This study examines the temporal fluctuations in the gut microbial composition across various life stages of *L. migratoria*. Additionally, we analyzed the digestibility of cellulose and hemicellulose in *L. migratoria* at different stages, while also measuring the enzyme activity of six bacterial species essential for cellulose degradation. These findings demonstrate that gut bacteria play a pivotal role in enhancing cellulose digestion in *L. migratoria*. Consequently, this investigation provides valuable data supporting the potential application of cellulolytic bacteria in biotechnology, such as biofuels and animal feed.

### Conclusion

Gut microbes of *L. migratoria* at different life stages are dynamic and correlate with their higher cellulose digestibility; the dominant gut bacteria have a strong cellulose-degrading capacity, and phytophagous insects have the potential to be developed into cellulose-degrading bioreactors.

## Data Availability

Metadata are stored in the SRA (BioProject PRJNA990365). The serial numbers are SAMN36267132–SAMN36267152.
